# Comparison of Adiposomal Lipids between Obese and Non-Obese Individuals

**DOI:** 10.3390/metabo14080464

**Published:** 2024-08-21

**Authors:** Mohamed Hussein, Imaduddin Mirza, Mohammed Morsy, Amro Mostafa, Chandra Hassan, Mario Masrur, Francesco M. Bianco, Subbaiah Papasani, Irena Levitan, Abeer M. Mahmoud

**Affiliations:** 1 Department of Pathology, University of Kentucky, Lexington, KY 40536, USA; msabry@uky.edu; 2 Department of Medicine, Division of Endocrinology, Diabetes, and Metabolism, College of Medicine, University of Illinois Chicago, Chicago, IL 60612, USA; mmirza24@uic.edu (I.M.); mmorsy3@uic.edu (M.M.); psubbaia@uic.edu (S.P.); 3 Department of Pharmacology, College of Medicine, University of Illinois Chicago, Chicago, IL 60612, USA; amost2@uic.edu; 4 Department of Surgery, College of Medicine, University of Illinois Chicago, Chicago, IL 60612, USA; chandrar@uic.edu (C.H.); mmasrur@uic.edu (M.M.); biancofm@uic.edu (F.M.B.); 5 Department of Medicine, Division of Pulmonary and Critical Care Medicine, University of Illinois Chicago, Chicago, IL 60612, USA; levitan@uic.edu; 6 Department of Kinesiology and Nutrition, College of Applied Health Sciences, University of Illinois Chicago, Chicago, IL 60612, USA

**Keywords:** adipose tissues, extracellular vesicles, adiposomes, obesity, lipidomics, phosphatidylcholine, lysophosphatidylcholine, ceramides, cardiometabolic disease, vascular function

## Abstract

Our recent findings revealed that human adipose tissues (AT)-derived extracellular vesicles (adiposomes) vary in cargo among obese and lean individuals. The main objective of this study was to investigate the adiposomal lipid profiles and their correlation with cardiometabolic risk factors. AT samples were collected from obese subjects and lean controls and analyzed for their characteristics and lipid content. In addition, we measured the correlation between adiposomal lipid profiles and body composition, glucose and lipid metabolic profiles, brachial artery vasoreactivity, AT arteriolar flow-induced dilation, and circulating markers such as IL-6, C-reactive protein, and nitric oxide (NO). Compared to lean controls, adiposomes isolated from obese subjects were higher in number after normalization to AT volume. The two major lipid classes differentially expressed were lysophosphatidylcholine/phosphatidylcholine (LPC/PC) and ceramides (Cer). All lipids in the LPC/PC class were several-fold lower in adiposomes from obese subjects compared to lean controls, on top of which were PC 18:2, PC 18:1, and PC 36:3. Most ceramides were markedly upregulated in the obese group, especially Cer d37:0, Cer d18:0, and Cer d39:0. Regression analyses revealed associations between adiposomal lipid profiles and several cardiometabolic risk factors such as body mass index (BMI), fat percentage, insulin resistance, arteriolar and brachial artery vasoreactivity, NO bioavailability, and high-density lipoproteins (HDL-C). We conclude that the ability of adiposomes from obese subjects to disrupt cardiometabolic function could be partly attributed to the dysregulated lipid cargo.

## 1. Introduction

Obesity is a significant health issue in the United States and all over the world. According to data released by the Centers for Disease Control and Prevention (CDC), from 2011 to 2018, obesity (BMI > 30 kg/m^2^) and severe obesity (BMI > 40 kg/m^2^) have climbed from 34.9% and 6.4% to 42.4% and 9.2% respectively [1]. This rise in obesity causes it to be a public health issue, which increases the risk of cardiovascular, metabolic, neoplastic diseases and several others [2]. However, the mechanisms by which obesity directly contributes to these conditions remain understudied.

A well-known factor caused by obesity is the buildup of dysfunctional adipose tissue. Adipose tissues have essential functions as an endocrine organ that produces several soluble mediators and hormones that regulate appetite, energy homeostasis, metabolism, insulin sensitivity, vascular function, and several other physiological processes [3]. Adipose tissues also release adipokines, which can promote inflammation and result in conditions such as Type 2 diabetes, atherosclerosis, kidney disease, and other vascular complications [2,4,5]. Aside from these soluble mediators and adipokines, adipose tissues can also produce extracellular vesicles (EVs), which we refer to as adiposomes [6]. Adiposomes are a newly appreciated mediator of several metabolic and cardiovascular diseases [7]. In the case of excess adiposity, adiposomes carry pro-inflammatory and other dysregulated metabolic cargo that impacts the body’s overall inflammatory and metabolic state [8].

Extracellular vesicles (EVs) have become a very intriguing science discipline. Previously, EVs were considered to be a form of cellular waste due to their small size and inactivity, but recent studies have linked EVs to significant roles in influencing cellular behavior and communication, suggesting their function may have been initially overlooked [9]. Adipose tissue is largely accepted to represent a key secretory organ that releases multiple bioactive molecules, and much of this secretome can be found in the adipose EVs (adiposomes) [10,11,12]. Our previous studies demonstrated the effect of adiposomes obtained from obese individuals in inducing endothelial cell dysfunction via disrupting cell surface caveolae and endothelial nitric oxide signaling [13,14]. Like other EVs, adiposomes are lipid-enclosed structures that carry RNA fragments, lipids, proteins, and other metabolites [15]. Our group and others have shown that the production of adiposomes increases in obesity and other metabolic diseases, contributing to inflammation and insulin resistance [13,14,16,17,18,19]. Yet, the cargo of adiposomes in obese individuals has remained to be explored. We hypothesize that adiposomes from obese individuals will contain dysregulated lipid content due to the dysregulated lipid metabolism in the adipose tissues, in contrast to those from healthy populations.

Compared to our information on the microRNAs and protein content of EVs, our understanding of the lipid composition of these vesicles and the role that lipids play in these vesicles is limited. Lipids are key molecular components of EVs, and their function is beginning to be recognized by the research community, which is becoming increasingly interested in these molecules. To contribute to filling this knowledge gap, we analyzed the composition of adiposomal lipids obtained from obese individuals and non-obese healthy controls. We also correlated these findings with anthropometric and cardiometabolic measurements. Therefore, we believe this research will facilitate the comprehension of biological messages that could be carried by adiposomes and potentially transferred to other cells and tissues in the body of obese individuals. The significance of this research stems from the crucial function of EVs in facilitating cellular communication, which renders them highly attractive as potential therapeutic agents, drug delivery systems, and disease biomarkers. Thus, harnessing the distinctive composition of adipose-generated EVs has the potential to open up new and enhanced avenues for diagnosing and treating obesity-related cardiometabolic diseases.

## 2. Materials and Methods

### 2.1. Participant Enrollment

Fat biopsies were acquired from 10 obese subjects and 10 lean controls while undergoing bariatric and elective hernia surgeries, respectively. These surgeries took place at the University of Illinois Hospital (Chicago, IL, USA). The inclusion criteria included males and females under 50 years old who were scheduled for the above-mentioned surgeries. Obese subjects had a body mass index (BMI) of more than 30 kg/m^2^, and lean subjects had a BMI of less than 25 kg/m^2^. Exclusion criteria included pregnant women, current smokers, current medications that modify lipid or metabolic profiles, previous bariatric surgery, cardiac, hepatic, and renal disease, cancer, and acute or chronic inflammatory conditions. All the protocols in this study were reviewed and deemed appropriate by University of Illinois Chicago (UIC)’s Institutional Review Board (IRB).

### 2.2. Cardiometabolic Risk Measurements

Anthropometric measurements of the study participants, including body weight and body mass index (BMI), were obtained. For body composition and fat and lean percentages, a DEXA (Dual X-ray absorptiometry) scan was used (iDXA, General Electric Inc. (Boston, MA, USA). Parameters of glucose metabolism, such as fasting glucose and fasting insulin levels, were measured following our previously published methods [20]. Hemoglobin A1c was evaluated via the Crystal Chem kit (Elk Grove Village, IL, USA). Insulin sensitivity was calculated using HOMA-IR (insulin resistance homeostasis model assessment). The equation for this index is as follows: fasting insulin (U/L) × fasting glucose (nmol/L)/22.5 [21]. In addition, measurements of lipid metabolism such as total cholesterol, low-density lipoproteins (LDL-C), HDL-C, and triglycerides were assessed via enzyme assays [4,20,22] (Roche Diagnostics, Indianapolis, IN, USA). Nitric oxide (NO) bioavailability in blood samples was evaluated using the Nitrate/Nitrite Colorimetric Assay Kit (Cayman Chemicals, Ann Arbor, MI, USA) [23,24,25]. Finally, circulating inflammatory biomarkers such as interleukin 6 (IL6) and C-reactive protein (CRP) were measured in plasma samples via High-Sensitivity Magnetic Luminex Performance Assays (R&D Systems, Minneapolis, MN, USA) and high-sensitivity ELISA assays (Crystal Chem, Elk Grove Village, IL, USA), respectively.

### 2.3. Adiposome Isolation

Fat biopsies were processed as we previously published [13]. Fat samples were cleaned from excess blood using sterile Medium 199 (Gibco, Waltham, MA, USA), cut with sterile scalpels into small pieces, and homogenized with a mixture of Type 1 collagenase (Worthington, Columbus, OH, USA) and 4% BSA (bovine serum albumin) in the medium. The homogenized fat was then filtered and centrifuged at 500 g. The floating mature adipocytes were collected and cultured on cell insert in the medium M199 supplemented with 1% penicillin/streptomycin and 5% exosome-free FBS (fetal bovine serum). Following 24–48 h, the media was collected and underwent several steps of centrifugation starting with 1000× *g* for 5 min, 15,000× *g* for 15 min, and passed through 0.45 mm filters. Finally, samples were ultracentrifuged to pellet adiposomes for 2 h at 150,000× *g*. Resuspended adiposomes were analyzed using a Nanoparticle Analyzer (NanoSight NS300, Malvern Instruments Ltd., Malvern, UK).

### 2.4. Adiposomal Lipid Isolation and Analysis

The extraction of lipids from lean and obese adiposomes was performed using the Folch method, as we previously described [13]. Agilent 6545 Q-TOF LC–MS system was used to analyze the extracted lipids from the adiposome samples. This system was paired with the Agilent Mass Hunter acquisition software (v. 12.1, Agilent Technologies, Santa Clara, CA, USA). For the lipid separation step, a 2.1 × 100 nm Agilent Poroshell C18, 2.7 μm column was sued, which was purchased from Agilent Technologies Inc. (Santa Clara, CA, USA). An Agilent 1290 UPLC system was used for this purpose, using a flow rate of 300 μL/min and a gradient of 70% mobile phase for a period that ranges from 0 to 1 min, followed by 86% mobile phase for a period ranging from 3.5 to 10 min, and finally 100% mobile phase for a period ranging from 11 to 17 min. All experimental runs used a consistent post-column equilibration time of 5 min. The following parameters were used in this experiment: 3000 voltage was applied to the VCap and 145 voltage for the fragmentor, the sheath gas flow rate was adjusted to be 12 L/min and temperature was set to 350 °C, the drying gas flow rate was set at 11 L/min, the gas temperature was maintained at 200 °C, and pressure of the nebulizer was adjusted to 35 psi. A lipid database was constructed using pooled samples. Lipids were scanned at a speed of 4 spectra/second, and the relative quantification of lipids was computed. The Mass Hunter acquisition program was used to execute a repeated MS/MS procedure.

### 2.5. Flow-Mediated Dilation (FMD)

The brachial artery’s FMD was assessed using an Aplio i900 ultrasound machine (Cannon Ultrasound Systems, Melville, NY, USA) [20]. The vascular probe was placed at a 60-degree angle 2 inches above the antecubital fossa, and a blood pressure cuff was placed around the middle of the forearm. The cuff was inflated to reach 220 mmHg for five minutes and then deflated. The arterial diameter was measured for 1 min before inflating the cuff (baseline; BSL) and for 5 min after deflating the cuff (reactive hyperemia (RH). Images and videos were captured and analyzed using the Automated Edge Detection software (Version 110.0.2; GE Medical Systems, Horten, Norway). The percentage flow-mediated dilation (FMD) was determined by subtracting the baseline diameter from the largest diameter observed during the hyperemia phase. This difference was then divided by the baseline diameter, multiplied by 100 as previously described in our publication [20,22,24,25,26].

### 2.6. Flow-Induced Dilation (FID) in Adipose Tissue Arterioles

Fat arterioles were dissected and isolated from fat samples, washed, cleaned of excess fat and connective tissues, and finally cannulated in an organ chamber as we previously described [4,5,22,23,24,25]. Briefly, arterioles were secured with a nylon ethilon suture around glass microcapillaries inside the organ chamber. Microcapillaries were then connected to a microfluidic system (tubing and reservoirs) containing Krebs buffer. These reservoirs were positioned in a specific way to expose the interior of arterioles to increasing pressure gradients ranging from 10 to 100 cm of water (cm H_2_O). The changes in the intraluminal diameter of the arterioles were visualized on a monitor after placing the chamber on an inverted Olympus microscope. At baseline, arterioles were exposed to endothelin (ET-1; 10^−6^ mol/L) to induce vasoconstriction. The vasodilation percentage was measured by normalizing the maximum arteriolar diameter at each pressure gradient to the diameter measured following the exposure to ET-1.

### 2.7. Statistical Analysis

Findings were reported as the mean ± standard error, and *p* < 0.05 was considered to be statistically significant. Differences between lean and obese subjects were examined using Student’s unpaired *t*-test. A statistically significant linear relationship between continuous variables was tested using a bivariate Pearson Correlation. The fold change in lipid species was calculated by dividing the expression level in obese by the expression level in lean control for each lipid species. The log2 fold change was calculated by taking the base 2 logarithm of the fold change. Analyses were performed using SPSS statistical software (version 26.0; SPSS Inc, Chicago, IL, USA) and R studio (v.4.4.1).

Principal Components Analysis (PCA), which allows the detection of significant trends in the samples, was conducted using all compounds in all samples. PCA was performed using R studio, starting by scaling the data to have mean zero and standard deviation one to ensure that each variable contributes equally to the PCA. This was followed by a prcomp function that uses Singular Value Decomposition (SVD) to compute principal components. Finally, a Biplot function combined a score plot of the samples and a loading plot of the variables. Clustering analysis was conducted to generate the heat map with a Hierarchical algorithm by clustering on conditions and compounds. The analysis started by scaling the data to have mean zero and standard deviation one, ensuring that each variable contributes equally to the clustering. Hierarchical Clustering was performed using the Ward method, and data matrices were visualized using the Heatmap function to aid in the identification of patterns and clusters. Significant compounds with *p*-value < 0.05 by Benjamini Hochberg False Discovery Rate (FDR) correction were imported into lipid pathway enrichment analysis (LIPEA) to carry out the pathway mapping after Fisher’s extract test enrichment.

For lipid analysis, the Lipid Annotator software (Agilent Technologies Inc., Santa Clara, CA, USA) created a fragmentation-based MS/MS library with m/z precursors and retention times for all identified lipids. The software was set to identify positive lipid species ([M + H]^+^, [M + Na]^+^, [M + NH_4_]^+^, [M + H − H_2_O]^+^, [M + Na − H_2_O]^+^, [M + NH_4_ − H_2_O]^+^), and Q-Score ≥ Profinder (vB.10.00, Agilent Technologies Inc., Santa Clara, CA, USA) processed raw LC–MS data. Molecular characteristics were retrieved for peaks ≥ 5000 counts in positive mode ([M + H]^+^, [M + Na]^+^, [M + NH_4_]^+^, [M + H − H_2_O]^+^, [M + Na − H_2_O]^+^, [M + NH_4_ − H_2_O]^+^) using an isotope model of common organic compounds (no halogens). Selected compounds were screened for absolute height ≥ 10,000 counts, quality score > 60, and the presence of two or more isotopes. The retention duration for each chemical was aligned to ±0.1 min using a mass accuracy window of ≤5.0 ppm, and peaks were integrated using the Agile integrator in Profinder software. Retention time and fragmentation matching were thoroughly checked for each integrated peak. After processing, the data file was exported into Mass Profiler Professional (v15.1, Agilent Technologies Inc., Santa Clara, CA, USA) for individual analysis. All chemical abundance measurements were baseline corrected to the median and normalized by IS lipid class. The nearest IS by retention time will be used if the class is not in IS.

## 3. Results

### 3.1. Characteristics of the Study Participants

Table 1 summarizes all the Demographic, anthropometric, metabolic, and cardiovascular risk parameters measured in the research subjects. Ten obese subjects and ten lean, healthy controls participated in the study (5 males and 5 females in each group). The average age for both groups was not statistically different. However, the average body weight and BMI were 1.2-fold higher in the obese subjects than the lean controls. Diastolic blood pressure was also higher in the obese subjects than in the lean controls. Metabolic parameters such as fasting plasma glucose and insulin levels were 50% and 120% higher in obese individuals compared to lean subjects, respectively. Subsequently, the insulin resistance index, HOMA-IR (homeostatic model assessment for insulin resistance), was significantly higher in the obese group (*p* = 0.0001). Finally, the lipid profile measurements, including triglycerides, total cholesterol level, and LDL-C (low-density lipoproteins), were markedly higher in the obese compared to lean subjects, while the HDL-C (high-density lipoprotein) was significantly lower in the former group (Table 1). Subjects were not administering any lipid-lowering agents or other medications known to alter lipid profiles at the time of recruitment.

**Figure 1:**
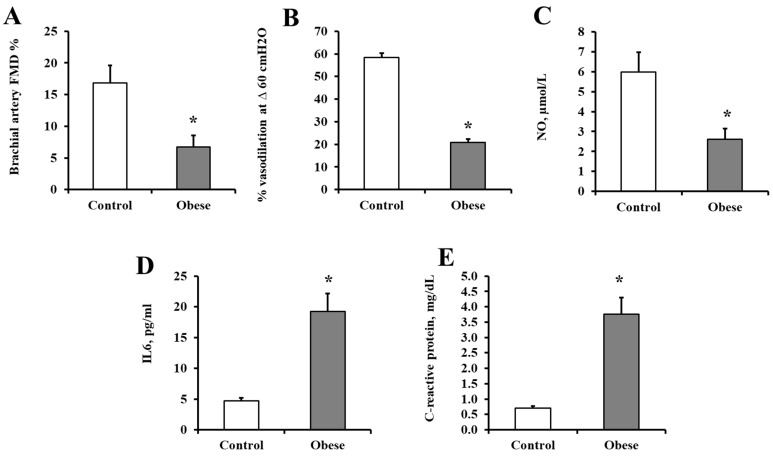
Vascular and systemic inflammatory measurements. Vascular measurements include brachial artery vasoreactivity (**A**), arteriolar flow-induced dilation (FID) (**B**), and nitric oxide (NO) (**C**) in obese subjects and lean, healthy controls (n = 10, each). Inflammatory measurements include IL6 (**D**) and CRP (**E**) in obese subjects and lean, healthy controls (n = 10, each). Data represents mean ± the standard error (SE); * *p*-value < 0.05.

In addition to the above-mentioned anthropometric and cardiometabolic risk measurements, vascular function was assessed via measurements of brachial artery vasoreactivity, arteriolar flow-induced dilation (FID), and circulating nitric oxide (NO) levels. For brachial artery reactivity, ultrasound imaging was used as described in the methods. The percentage of vasodilation of the brachial artery following reactive hyperemia was significantly lower in the obese subjects compared to lean controls (85% lower, *p* = 0.004; Figure 1A). FID measured in isolated arterioles from visceral adipose tissue (VAT) samples exhibited less vasoreactivity in response to the increasing pressure gradient. Figure 1B shows FID at the pressure gradient 60 cm H_2_O, reflecting the human body’s physiological flow rate and intraluminal pressure. Under these conditions, arteriolar FID was found to be impaired in the obese group compared to lean, healthy controls (94% lower, *p* = 3.01 × 10^−12^). Similarly, circulating NO levels were much lower in the obese group compared to lean controls (80% lower, *p* = 0.004; Figure 1C). For systemic inflammation, IL6 and CRP were measured. Both biomarkers were several folds higher in the obese subjects than lean controls (*p* = 4.9 × 10^−5^ and 1.4 × 10^−5^, respectively; Figure 1D,E).

### 3.2. Adiposomal Characterization

**Figure 2:**
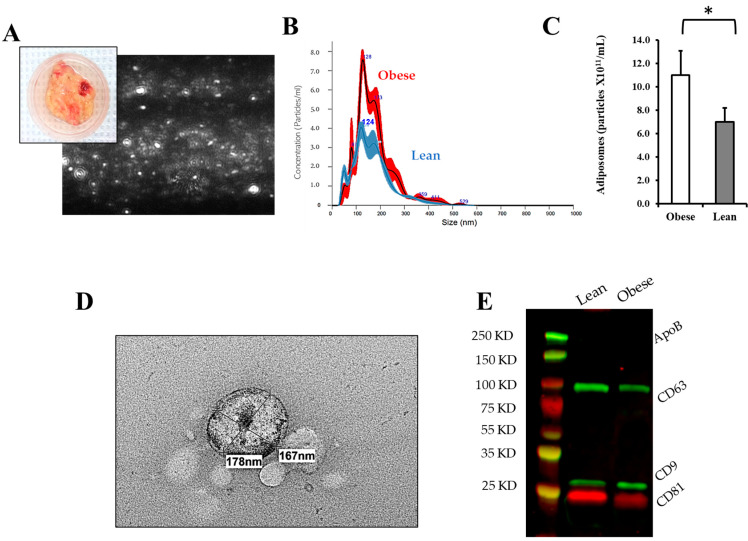
Adiposome isolation and characterization. (**A**) A representative image of adipose tissue samples and a screenshot of adiposome particles quantified via the nanoparticle-tracking analysis device (Nanosight NS300). (**B**) A representative chart of the quantified adiposomes from obese and lean subjects. (**C**) A bar chart representing the mean ± SE of adiposome particles isolated from obese subjects and lean controls (n = 10 each). (**D**) Transmission Electron Microscopy (TEM) image of the isolated adiposomes. (**E**) Representative Western blots for analyzing adiposome-extracted proteins for tetraspanins (CD9/DC63/CD81). * *p*-value < 0.05.

Adiposomes were extracted from visceral fat samples collected from lean and obese participants using sequential steps of tissue homogenization, centrifugation, and filtration as described in the methods. Adiposomes were characterized for their size and number in each subject using NanoSight NS300 Analyzer for nanoparticle-tracking analysis (NTA) (Figure 2A). The diameter of adiposomes ranged from 50 nm to 300 nm and was not statistically different between the two groups. However, the number of adiposomes was significantly elevated in the obese subjects (11.2 × 10^11^ particles/mL) compared to lean controls (7.1 × 10^11^ particles/mL, *p* = 0.005) (Figure 2B,C). The vesicular club-shaped structure of adiposomes was also confirmed using electron microscopy (Figure 2D). The protein content of the adiposomes was analyzed to verify their extracellular vesicular nature. Figure 2E shows that adiposomes express tetraspanins (CD9, CD81, and CD63), which are extracellular vesicle-characterizing proteins. Protein analysis also verified the lack of Apolipoprotein B contamination.

### 3.3. Adiposomal Lipid Content

**Figure 3:**
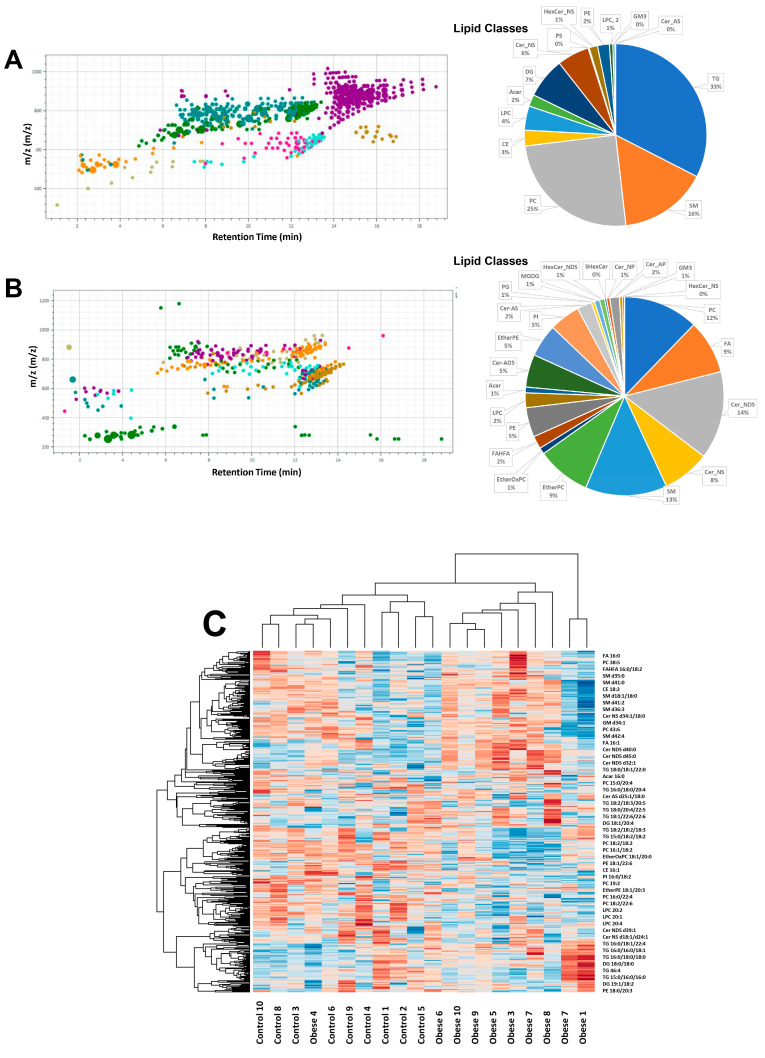
Lipid analysis in adiposomes via mass spectrometry. Lipid classes were identified in the positive mode (**A**) and the negative mode (**B**). Heat map clustering of samples and compounds; brown is for increasing and blue is for decreasing expression (**C**).

A non-targeted mass spectrometry analysis was performed using the Q-TOF LC–MS system to analyze the relative abundance of different lipids in adiposomes isolated from VAT. We detected 562 lipids in reversed-phase LC/MS/MS in the positive (Figure 3A) and negative modes (Figure 3B). Figure 3C shows a heat map representation (log base 2 of normalized values) of the 562 lipid species that belong to 22 major lipid classes found in VAT adiposomes.

**Figure 4:**
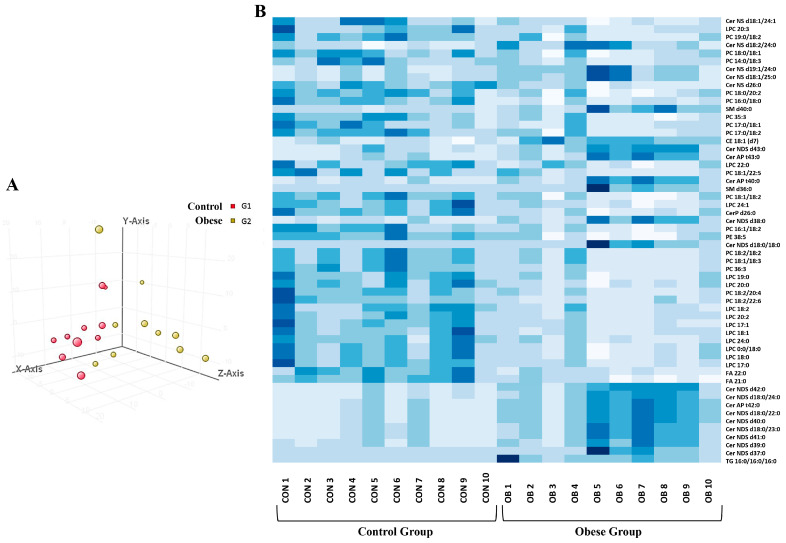
Lipid analysis in adiposomes isolated from obese and lean control subjects. (**A**) Principal Component Analysis (PCA) of the identified lipids in isolated adiposomes. (**B**) Heat map representation of adiposomal lipids that were statistically different between obese and lean control subjects.

It can be inferred from the scatterplot of the first two principal components that there is a reasonable distinction between the obese and lean healthy phenotypes. This suggests that the lipid content of adiposomes is informative enough to differentiate between the two groups (Figure 4A). Following correction for multiple tests, the results, which had been adjusted to internal standards, revealed significant differences in the abundance of 56 lipid species between adiposomes obtained from obese individuals and lean controls, as illustrated in the heatmap presented in Figure 4B.

**Figure 5:**
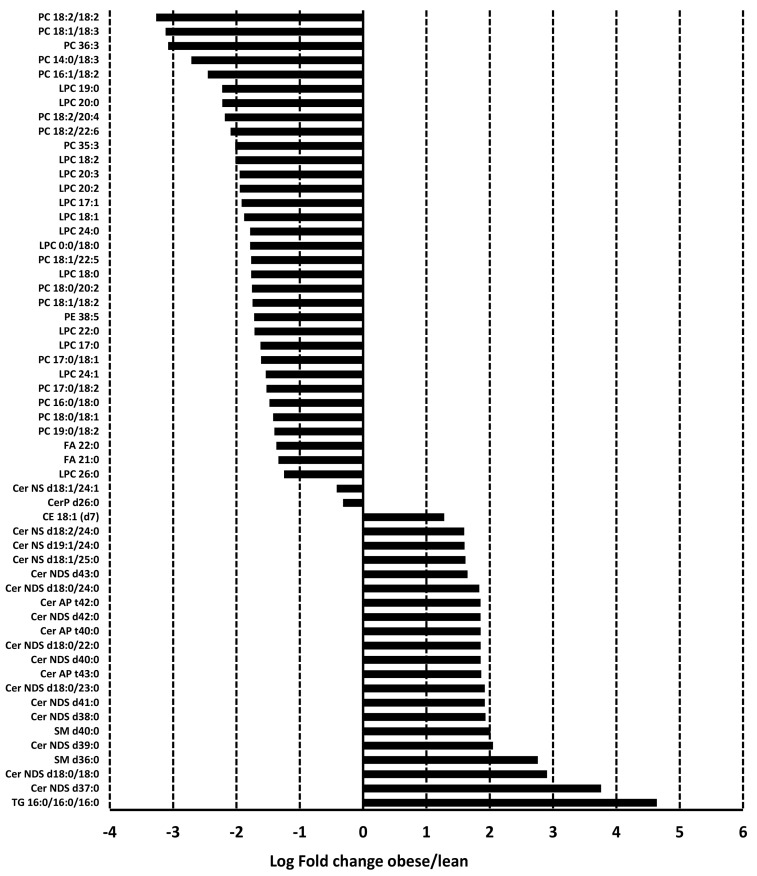
Differential expression of adiposomal lipids. The average significant fold changes (*q* < 0.05) observed in adiposomal lipids from obese and lean individuals (n = 10, each).

The two major lipid classes that were differentially expressed are lysophosphatidylcholine/phosphatidylcholine (LPC/PC) and ceramides (Cer). Other lipid classes included triglycerides, sphingomyelins, and fatty acids. All lipids in the LPC/PC class were significantly downregulated in adiposomes from obese subjects compared to lean controls (Figure 5 and Supplementary Table S1). Five of these LPCs/PCs were between 5.5 and 9.6 folds higher in the lean subjects compared to obese subjects, including PC 18:2/18:2, PC 18:1/18:3, PC 36:3, PC 14:0/18:3, and PC 16:1/18:2. Six LPCs/PCs were more than four folds higher in the lean control group including LPC 19:0, LPC 20:0, PC 18:2/20:4, PC 18:2/22:6, PC 35:3, and LPC 18:2. The rest of the LPCs/PCs were between 2.4 and 3.9 folds higher in the lean control group than obese subjects.

Regarding ceramides, 17 of them were upregulated in the obese group. On top of these ceramides are Cer d37:0 (14 folds), Cer d18:0/18:0 (7.5 folds), and Cer d39:0 (4 folds). The rest of the ceramides were 3 to 3.8-fold higher in obese subjects than lean controls. Two subspecies of ceramides (CerP d26:0, Cer d18:1/24:1) were downregulated yet with borderline significance Figure 5 and Supplementary Table S2). Other differentially expressed lipid species included two fatty acids, FA 22:0 and FA 21:0, that were downregulated in the obese subjects compared to lean controls (2.5-fold lower, *q* = 0.02). Also, Triglyceride TG 16:0-16:0-16:0 was 25-fold higher in obese subjects than lean controls (*q* = 0.0002). Finally, two sphingomyelin lipid residues, SM d36:0 and SM d40:0, were higher in obese individuals than lean controls (6.8 and 4-fold higher, respectively) (Figure 5 and Supplementary Table S1). No significant differences were observed between males and females or different racial/ethnic groups.

### 3.4. Relationship between the Adiposomal LPCs and Cardiometabolic Risk Factors

**Figure 6:**
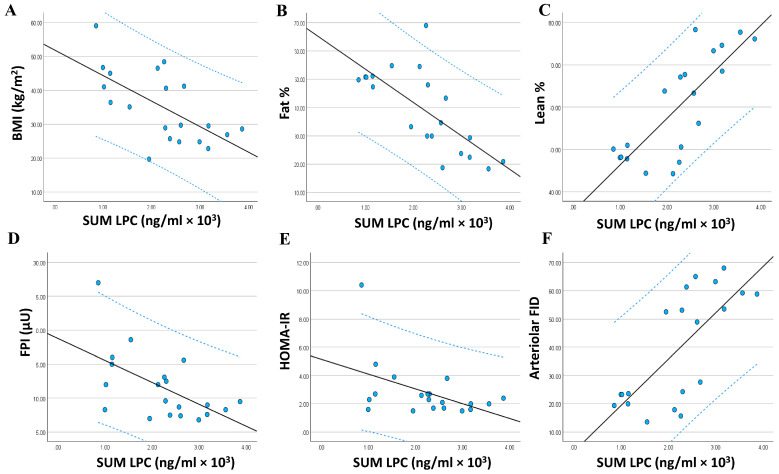
Association between adiposomal lysophosphatidylcholine (Sum LPC) and cardiometabolic risk factors. The association between Adiposomal LPC in obese and lean individuals and body mass index (BMI) (**A**), body fat percentage (**B**), body lean percentage (**C**), fasting plasma insulin (FPI) (**D**), homeostatic model assessment for insulin resistance (HOMA-IR) (**E**), and arteriolar flow-induced dilation (FID) (**F**). Data are presented as a scatter plot with a fitted regression line (solid line) and 95% confidence intervals (dotted line).

To investigate the association between adiposomal LPC/PC levels and cardiometabolic risk factors, all LPC/PC lipids were summed (Sum LPC), and a regression analysis was performed (Figure 6A–F). The regression analysis revealed significant associations between Sum LPC and several cardiometabolic risk factors on top of which are BMI (*r* = −0.635, *p* = 0.001), fat percentage (*r* = −0.715, *p* < 0.0001), lean percentage (*r* = 0.794, *p* < 0.0001), fasting insulin (*r* = −0.574, *p* = 0.004), HOMA-IR (*r* = −0.469, *p* = 0.019), and arteriolar flow-induced dilation (FID) (*r* = 0.733, *p* < 0.0001) (Figure 6). Significant associations were also observed between individual LPCs and other cardiovascular factors such as brachial artery flow-mediated dilation (FMD), nitric oxide (NO) bioavailability, and systolic blood pressure (SBP) and metabolic factors such as fasting glucose and high-density lipoproteins (HDL-C). These associations were summarized in Table 2, Table 3 and Table 4.

### 3.5. Relationship between the Adiposomal Ceramides and Cardiometabolic Risk Factors

**Figure 7:**
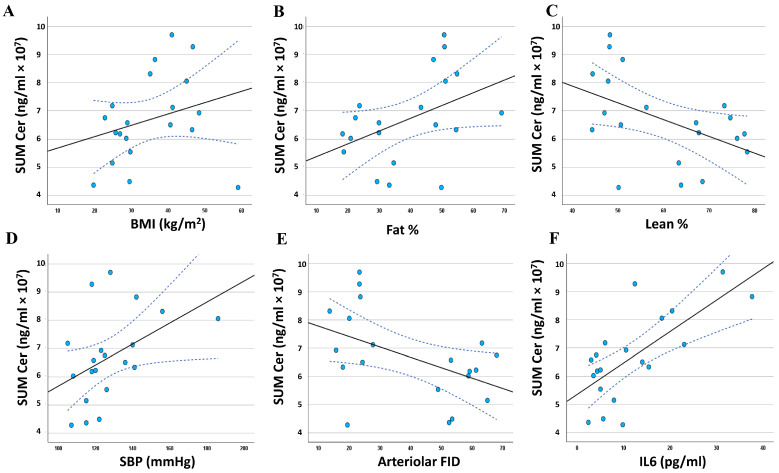
Association between adiposomal ceramides (Sum Cer) and cardiometabolic risk factors. The association between Adiposomal ceramides in obese and lean individuals and body mass index (BMI) (**A**), body fat percentage (**B**), body lean percentage (**C**), systolic blood pressure (SBP) (**D**), arteriolar flow-induced dilation (FID) (**E**), and interleukin 6 (IL6) (**F**). Data are presented as a scatter plot with a fitted regression line (solid line) and 95% confidence intervals (dotted line).

To investigate the association between adiposomal ceramide levels and cardiometabolic risk factors, all ceramides were summed (Sum Cer), and a regression analysis was performed (Figure 7A–F). The regression analysis revealed significant associations between Sum Cer and several cardiometabolic risk factors on top of which are fat percentage (*r* = 0.427, *p* = 0.030), lean percentage (*r* = −0.470, *p* =0.018), systolic blood pressure (SBP) (*r* = 0.453, *p* = 0.022), arteriolar flow-induced dilation (FID) (*r* = −0.470, *p* =0.018), and interleukin 6 (IL6) (*r* = 0.704, *p* < 0.0001). These associations are summarized in Table 5 and Table 6.

Significant associations were also observed between A1C and individual ceramides such as Cer d18:1/25:0 (*r* = 0.348, *p* = 0.038), Cer d19:1/24:0 (*r* = 0.377, *p* = 0.036), and Cer d18:2/24:0 (*r* = 0.399, *p* = 0.019). These three ceramides showed negative correlations with circulating nitric oxide (NO) (*r* = −0.484, −0.484, and −0.703, *p* = 0.015, 0.015, and 0.000, respectively) and positive correlations with C-reactive protein (*r* = 0.502, 0.498, and −0.450, *p* = 0.012, 0.013, and 0.023, respectively). Cer d18:2/24:0 also correlated positively with LDL-C (*r* = 0.476, *p* = 0.017) and negatively with HDL-C (*r* = −0.403, *p* = 0.039).

### 3.6. Pathway Analysis

**Figure 8:**
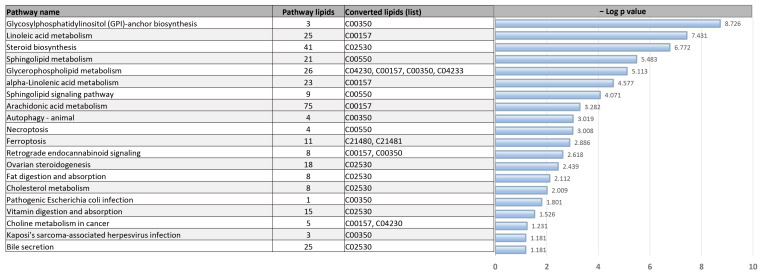
Pathway analysis of adiposomal lipids differentially expressed between lean and obese individuals.

The pathway analysis was conducted by LIpid Pathway Enrichment Analysis (LIPEA) online software (v.3.0, Biomedical Cybernetics Group, Biotechnology Center (BIOTEC), Technische Universität Dresden, Dresden, Germany) using the lipids that were significantly different between the obese and lean groups (*q* value < 0.05 or FC > 2). The differentially expressed lipids in the adiposomes from lean and obese individuals were found to be enriched in metabolic pathways such as linoleic acid metabolism, sphingolipid metabolism, cholesterol metabolism, and others. These lipids were also enriched in cellular homeostasis pathways such as autophagy, steroid biosynthesis, bile secretion, and others. These pathways are summarized in Figure 8 below.

## 4. Discussion

The adipose tissue represents a key secretory organ that releases multiple bioactive molecules, including extracellular vesicles (adipose tissue EVs; adiposomes) [10,11,12]. Recently, adiposomes have surfaced as innovative conduits for intercellular and interorgan communication through transporting bioactive cargo, including but not limited to miRNAs, RNAs, lipids, and proteins. Studies by our group and others demonstrated the role of adiposomes in reciprocal signaling between adipocytes and other cells, including endothelial cells and macrophages [13,27]. An estimated two thirds of the volume of adiposomes is composed of lipids, which are crucial signaling molecules [28]. However, in contrast to protein and nucleic acid, the lipid content in EVs has received far less attention, and its relationship with cardiometabolic function has not been adequately studied.

Lipids are integral constituents of cell membranes and are crucial in essential cellular functions, such as metabolism and signaling. Lipidomics has emerged as a significant field in medical and therapeutic sciences. This method has been employed in studying obesity to understand the physiologic and diagnostic significance of lipid alterations in obesity and their association with cardiometabolic risk. A significant component of metabolic syndrome is dyslipidemia. While this typically manifests as elevated levels of circulating triglycerides, low-density lipoproteins, and free fatty acids, adiposomal composition and quantity of lipids may also be modified, further contributing to dyslipidemia. Adiposomes may play a role in obesity-associated cardiometabolic diseases since they eventually circulate in the blood, interact with endothelial cells, and affect various metabolic and vascular organs and tissues. Therefore, to gain more insight into the adiposomal cargo, we conducted a lipidomic analysis, which we believe is the first to compare adiposomes from obese and lean individuals and link these lipid profiles to clinical and physiological measurements that reflect the cardiometabolic risk.

Our lipidomic analysis of adiposomes obtained from lean and obese individuals demonstrated that phosphatidylcholine (PC), lysophosphatidylcholine (LPC), and ceramides (Cer) comprised the top differential lipids. The PC/LPC group of lipids was consistently and markedly downregulated in the obese group compared with the lean subjects. Ceramides were significantly upregulated except for Cer d26:0 and Cer d18:1/24:1, which were slightly downregulated with borderline significance. Of the 56 differential lipids, only six belonged to other lipid species, including fatty acids, triglycerides, and sphingomyelin.

Although we found significant differences in the adiposomal lipidomic profile between obese subjects and lean controls, the most striking observation was the consistent reduction in 30 PC/LPC lipid species, which constituted more than 50% of the differential lipid content. Our data are consistent with previous studies by Barber et al. [29], showing a marked reduction in LPC species in plasma from obese and obese diabetic individuals compared to lean controls. Indeed, several studies have shown a strong inverse association between BMI and plasma levels of PC species such as PC 30:0, PC 34:2, PC 34:3, PC 36:2, PC 38:4, PC 40:6, PC 42:3, PC 42:4, PC 44:4, PC 44:5, and PC 44:6 [30,31,32,33,34,35]. Also, lower circulating levels of PC 32:0, PC 35:4, PC 36:2, and PC 38:5 have been reported in obese and insulin-resistant individuals [36]. Negative associations were evident in most studies that tested the relationship between BMI and LPC species, mainly LPC 15:0, LPC 16:0, LPC 17:0, LPC 18:0, LPC 18:1, LPC 18:2, LPC 20:0, LPC 20:1, LPC 20:2, and LPC 20:4 [29,30,31,32,33,34,36,37,38,39,40]. These LPCs were primarily examined in the circulation in the research stated above. Our current investigation demonstrated that they were similarly decreased in the adiposomes of obese individuals compared to lean controls.

Nevertheless, conflicting results have been reported showing positive correlations between BMI or visceral fat mass and PC species such as PC 18:0, PC 22:6, PC 15:0, PC 20:4, PC 28:1, PC 32:1, PC 38:3, PC 38:4, PC 32:1, PC 32:2, PC 36:3, PC 38:3, PC 40:4, and PC40:6 [30,31,33,34,35,37]. Similarly, positive associations have been reported between BMI and LPCs, such as LPC 14:0, LPC 16:0, LPC 22:5, LPC 22:6, LPC 16:1, and LPC 20:3 [31,33,34]. Interestingly, Bellot et al. [41] observed a distinct pattern of association between LPC and BMI when they divided obese individuals into metabolically healthy and metabolically ill groups. For instance, metabolically healthy individuals exhibited a positive correlation with PC 32:1 and PC 38:3, but were negatively associated with LPC 18:1, LPC 18:2, and LPC 18:0. Conversely, the metabolically unhealthy group exhibited an increase in LPC 16:1, PC 32:2, and PC 34:2, and a decrease in LPC 18:1, LPC 18:2, and PC 34:3. These data may partially explain the inconsistent reports of PC and LPC levels in obesity, which may also encourage future studies to consider metabolic health status as one of the critical variables that could potentially influence the circulating levels of these lipid species.

Similar inconsistencies in PC/LPC plasma levels in response to weight loss studies have been reported. While Schwab et al. [42] reported a lack of any significant effect of a 33-week weight loss intervention on plasma levels of LPCs, Heimerl et al. [39] reported substantial increases in plasma LPC levels as the BMI was lowered during a 55-week weight loss intervention. These findings might indicate the need for longer weight loss interventions to observe significant changes in PC/LPC levels in obese individuals. Yet, the discrepancy in the current literature is not fully understood, and several factors might contribute to these mixed findings, including genetic and dietary intake factors that simultaneously contribute to the development of obesity and altered PC synthesis [29,43,44].

PCs are the major lipid component of all cell membranes and subcellular organelles, and they are converted to LPCs via the action of phospholipase A2. LPCs are an integral component of oxidized LDL-C and contribute to the development and progression of atherosclerosis. Previous studies have associated higher levels of LPCs with mitochondrial dysfunction, impaired energy metabolism, vascular dysfunction, atherogenesis, and induced inflammation and oxidative stress [40,45]. Within this framework, PCs and their metabolites appear to be implicated in the development of cardiometabolic diseases. On the other hand, other studies highlighted the importance of PCs and LPCs in vital biological processes such as lipoprotein formation and stability [46,47], VLDL-C secretion by the liver [48,49], lipid droplet formation in the adipose tissue [50], key mitochondrial functions [51], metabolite uptake by the intestinal brush border [52], and skeletal muscle regeneration and contraction [53], among others. Therefore, it is clear that PC and LPC levels should be tightly regulated to maintain homeostasis and that either abnormally high or abnormally low levels could influence metabolic and vascular functions.

In previous research, low PC and LPC levels have been shown to correlate with impaired vascular and metabolic function and induce inflammation. For example, previous studies have shown LPCs to promote vasorelaxation via inhibiting endothelin-1 release or by inducing eNOS and cyclooxygenase-2 expression in endothelial cells, favoring the vasodilative over vasoconstrictors pathways [54,55,56,57,58,59]. Previous studies also revealed that LPCs enhanced the antioxidative capability of HDL-C and shielded LDL-C from oxidation [60]. In addition, LPC intake increased coronary blood flow and reduced mean arterial pressure and total vascular resistance in rodents [61]. LPCs also inhibited platelet aggregation and attenuated coagulation and smooth muscle cell proliferation in atherosclerotic lesions [62]. LPCs were found to possess antioxidative activity by inducing superoxide mutase [63]. Furthermore, they bind C-reactive protein (CRP) and subsequently delay macrophage activation and atherosclerosis development [64,65]. In agreement with these studies, our current investigation demonstrated positive correlations between PC/LPC levels and nitric oxide availability, brachial artery flow-mediated dilation, adipose tissue arteriolar vasoreactivity, and plasma HDL-C levels. We also observed inverse correlations between several PCs and LPCs and fasting plasma glucose and insulin, as well as the homeostatic model assessment for insulin resistance (HOMA-IR) and inflammatory markers such as IL6 and C-reactive protein (CRP).

In addition to the above-mentioned clinical studies, experimental investigations have established a connection between diet-induced obesity and decreased circulating LPC levels. Barber et al. [29] demonstrated that mice fed a high-fat diet for 12 weeks experienced reduced plasma and metabolic tissue levels of LPCs. The authors attributed this phenomenon to increased enzyme activity that breaks down PCs and LPCs. Their findings were further corroborated by the decreased levels of plasma LPCs observed in both obese non-diabetic and obese diabetic individuals.

While our findings provide evidence that obesity is linked to changes in adiposomal PC/LPC profiles, we cannot exclude the impact of nutrition. Choline, the precursor for PC synthesis, is an essential human nutrient [66]. While it can be synthesized endogenously, this is not an adequate source for the body’s needs. Therefore, choline must be obtained from choline-rich diets, particularly animal products, such as meat, dairy, eggs, and liver [67,68]. Previous studies have established the link between choline intake and PC circulating levels [69]. In our research, we could not draw any conclusions about the relationship between diet and adiposomal PC/LPC levels as we do not have accurate measurements of dietary choline intake. Nevertheless, future research must investigate this relationship.

Aside from the altered PC/LPC profiles in our cohort, obesity was associated with several-fold increases in saturated ceramide species such as Cer d18:0, Cer d19:0, Cer d37:0, Cer d39:0, Cer d40:0, Cer d42:0, and several others. The results of our investigation align with recent reports that showed higher levels of plasma ceramides in obese, insulin-resistant, and diabetic individuals [70]. However, our work is the first to show an increase in ceramide content in adiposomes specifically. Elevated ceramide levels have been linked to inflammation, oxidative stress, and endothelial dysfunction. Thus, the higher amount of ceramide carried by released adiposomes may negatively impact the function of distant cells and tissues, such as endothelial cells, into which these adiposomes are incorporated. Our prior mechanistic studies confirmed that adiposomes from obese patients can fuse with cultured endothelium cells, resulting in detrimental effects on their structure and function. More specifically, we have shown the adverse effects of obese adiposomes on cell surface caveolae, eNOS signaling, and membrane integrity [13,71]. The present work provides further evidence by demonstrating associations between dysregulated lipids carried by adiposomes and vascular dysfunction, which is characterized by poor dilatation of the brachial artery, reduced vasoreactivity in arterioles, elevated systolic blood pressure, and decreased availability of nitric oxide. The presence of ceramide cargo was also correlated with circulating inflammatory biomarkers, such as CRP and IL6.

Lipids are involved in numerous biological pathways and may serve as an indicator of the potential complications that may arise in obese individuals. The results of this study appear to substantiate the hypothesis that there are variations in adiposomal lipid composition between obese and non-obese individuals. These variations may contribute to the development of complications in obese individuals. However, the study’s findings are subject to certain limitations and require careful interpretation. The small sample size is the most significant of these limitations, as it may have restricted our capacity to identify differences between groups. Consequently, additional research utilizing large cohorts is necessary to determine whether there are any disparities in the adiposomal LPC profile between men and women and among individuals from various racial/ethnic backgrounds.

Replicating this study with a larger sample size might potentially produce slightly divergent outcomes from those obtained in this study. The rationale for this is that obese persons may have comorbidities that could influence the results in a different direction. According to the principal component analysis shown in Figure 4, the lipid profile of obese individuals exhibited greater variability than healthy individuals. This variability could be attributed to a myriad of obesity-related complications. Finally, the influence of diet on the results collected is an area that has not been thoroughly investigated in this study. Long-term malnutrition diets may be the direct cause of altered lipid composition rather than obesity itself. Consequently, it would be advantageous to conduct future research that regulates participants’ diets or collect comprehensive questionnaires on dietary behavior to differentiate the influence of obesity from the impact of nutritional style on adiposomal lipid composition.

## 5. Conclusions

In conclusion, our findings indicate that obesity substantially modifies the adiposomal lipidome. The most remarkable finding was the significant decrease in plasma LPC species among obese individuals. LPCs have long been perceived as detrimental; however, recent mechanistic studies have demonstrated their beneficial effects on oxidative stress, inflammation, and cardiometabolic function. Furthermore, these studies were supported by clinical findings indicating the reduction in circulating LPC in obese and diabetic humans. This concept is especially significant due to the recent increase in awareness of the beneficial metabolic effects of lipids. According to the results of this investigation, there is a necessity for a more profound comprehension of the mechanistic relationship between low levels of PCs and LPCs and obesity or obesity-related complications. The results of the present investigation may offer insight into the mechanism by which obesity increases the risk of cardiometabolic diseases. Moreover, identifying distinct adiposomal lipid profiles that are associated with cardiometabolic dysfunctions in obese individuals could serve as a diagnostic tool for the detection of cardiometabolic risk or lead to the creation of new drugs or therapeutic interventions.

## Figures and Tables

**Table 1:** Anthropometric and cardiometabolic characteristics of the participants.

Variable	Lean (n = 10, 5 Females)	Obese (n = 10, 5 Females)	*p*-Value
Age, y	36 ± 5	35 ± 7	NS
Anthropometric			
Weight, kg	69.5 ± 3.1	152.4 ± 9.7	<0.0001
BMI, kg/m^2^	22.4 ± 1.4	47.8 ± 5.9	<0.0001
Cardiometabolic			
FPI, µU/mL	6.4 ± 0.3	14.2 ± 1.9	0.0007
FPG, mg/dL	85 ± 7	127 ± 10	0.0029
HOMA-IR	2.2 ± 0.3	5.2 ± 0.5	0.0001
HbA1c, %	5.4 ± 0.2	5.7 ± 0.3	NS
Chol, mg/dL	147.5 ± 5.5	192.0 ± 11	0.0020
LDL-C, mg/dL	85.1 ± 4.6	111.3 ± 6.1	0.0030
HDL-C, mg/dL	57.0 ± 3.7	32.0 ± 4.7	0.0006
Trig, mg/dL	91.6 ± 4.2	127.0 ± 5.2	<0.0001
HR, bpm	75 ± 4	80 ± 5	NS
SBP, mmHg	118 ± 2	122 ± 4	NS
DBP, mmHg	75 ± 2	84 ± 2	0.0052

BMI, body mass index; Chol, cholesterol; DBP, diastolic blood pressure; FPG, fasting plasma glucose; FPI, fasting plasma insulin; HbA1c, glycosylated hemoglobin; HDL-C, high-density lipoprotein; HOMA-IR, homeostatic model assessment for insulin resistance; HR, heart rate; LDL-C, low-density lipoprotein; NS, not significant; SBP, systolic blood pressure; Trig, triglycerides.

**Table 2:** Association between adiposomal PC/LPC and anthropometric measurements.

	BMI		Fat %		Lean %	
	r	*p*-Value	r	*p*-Value	r	*p*-Value
LPC 17:0	−0.552 **	0.006	−0.764 **	0.000	0.804 **	0.000
LPC 18:0	−0.562 **	0.005	−0.721 **	0.000	0.777 **	0.000
LPC 18:0	−0.569 **	0.004	−0.728 **	0.000	0.785 **	0.000
LPC 24:0	−0.604 **	0.002	−0.698 **	0.000	0.769 **	0.000
LPC 18:1	−0.559 **	0.005	−0.731 **	0.000	0.760 **	0.000
LPC 17:1	−0.535 **	0.008	−0.740 **	0.000	0.784 **	0.000
LPC 20:2	−0.552 **	0.006	−0.735 **	0.000	0.806 **	0.000
LPC 18:2	−0.631 **	0.001	−0.793 **	0.000	0.830 **	0.000
PC 18:2/22:6	−0.614 **	0.002	−0.731 **	0.000	0.747 **	0.000
PC 18:2/20:4	−0.543 **	0.007	−0.709 **	0.000	0.769 **	0.000
LPC 20:0	−0.587 **	0.003	−0.729 **	0.000	0.785 **	0.000
LPC 19:0	−0.550 **	0.006	−0.702 **	0.000	0.791 **	0.000
PC 36:3	−0.576 **	0.004	−0.696 **	0.000	0.768 **	0.000
PC 18:1/18:3	−0.552 **	0.006	−0.683 **	0.000	0.760 **	0.000
PC 18:2/18:2	−0.544 **	0.007	−0.670 **	0.001	0.749 **	0.000
PC 16:1/18:2	−0.590 **	0.003	−0.580 **	0.004	0.660 **	0.001
LPC 24:1	−0.491 *	0.014	−0.624 **	0.002	0.724 **	0.000
PC 18:1/18:2	−0.564 **	0.005	−0.606 **	0.002	0.700 **	0.000
PC 18:1/22:5	−0.525 **	0.009	−0.522 **	0.009	0.596 **	0.003
LPC 22:0	−0.329	0.078	−0.652 **	0.001	0.713 **	0.000
PC 17:0/18:2	−0.559 **	0.005	−0.578 **	0.004	0.656 **	0.001
PC 17:0/18:1	−0.551 **	0.006	−0.606 **	0.002	0.623 **	0.002
PC 35:3	−0.490 *	0.014	−0.609 **	0.002	0.692 **	0.000
PC 16:0/18:0	−0.578 **	0.004	−0.659 **	0.001	0.722 **	0.000
PC 18:0/20:2	−0.574 **	0.004	−0.608 **	0.002	0.687 **	0.000
LPC 26:0	−0.712 **	0.000	−0.684 **	0.000	0.720 **	0.000
PC 14:0/18:3	−0.515 *	0.010	−0.491 *	0.014	0.520 **	0.009
PC 18:0/18:1	−0.650 **	0.001	−0.623 **	0.002	0.649 **	0.001
PC 19:0/18:2	−0.537 **	0.007	−0.582 **	0.004	0.691 **	0.000
LPC 20:3	−0.521 **	0.009	−0.660 **	0.001	0.688 **	0.000
LPC 15:0	−0.535 **	0.008	−0.604 **	0.002	0.651 **	0.001
SUM LPC	−0.635 **	0.001	−0.715 **	0.000	0.794 **	0.000

* for *p* values < 0.05 and ** for *p* values < 0.01.

**Table 3:** Association between adiposomal PC/LPC and cardiovascular measurements.

	SBP		NO		FMD		FID	
	r	*p*-Value	r	*p*-Value	r	*p*-Value	r	*p*-Value
LPC 17:0	−0.306	0.095	0.352	0.064	0.289	0.108	0.687 **	0.000
LPC 18:0	−0.281	0.115	0.515 *	0.010	0.341	0.070	0.684 **	0.000
LPC 18:0	−0.289	0.108	0.525 **	0.009	0.355	0.062	0.692 **	0.000
LPC 24:0	−0.223	0.172	0.523 **	0.009	0.388 *	0.046	0.722 **	0.000
LPC 18:1	−0.314	0.089	0.459 *	0.021	0.254	0.140	0.691 **	0.000
LPC 17:1	−0.347	0.067	0.350	0.065	0.254	0.140	0.684 **	0.000
LPC 20:2	−0.334	0.075	0.351	0.065	0.381 *	0.049	0.657 **	0.001
LPC 18:2	−0.332	0.077	0.397 *	0.042	0.475 *	0.017	0.659 **	0.001
PC 18:2/22:6	−0.357	0.061	0.355	0.062	0.233	0.161	0.707 **	0.000
PC 18:2/20:4	−0.367	0.056	0.309	0.093	0.359	0.060	0.656 **	0.001
LPC 20:0	−0.273	0.122	0.479 *	0.016	0.419 *	0.033	0.704 **	0.000
LPC 19:0	−0.360	0.059	0.378 *	0.050	0.381 *	0.049	0.711 **	0.000
PC 36:3	−0.421 *	0.032	0.368	0.055	0.599 **	0.003	0.684 **	0.000
PC 18:1/18:3	−0.411 *	0.036	0.350	0.065	0.602 **	0.002	0.650 **	0.001
PC 18:2/18:2	−0.400 *	0.040	0.332	0.076	0.588 **	0.003	0.644 **	0.001
PC 16:1/18:2	−0.465 *	0.019	0.314	0.088	0.259	0.135	0.707 **	0.000
LPC 24:1	−0.280	0.116	0.531 **	0.008	0.406 *	0.038	0.695 **	0.000
PC 18:1/18:2	−0.371	0.053	0.353	0.064	0.531 **	0.008	0.662 **	0.001
PC 18:1/22:5	−0.368	0.055	0.281	0.115	0.081	0.367	0.622 **	0.002
LPC 22:0	−0.363	0.058	0.327	0.080	0.435 *	0.028	0.588 **	0.003
PC 17:0/18:2	−0.376	0.051	0.215	0.181	0.413 *	0.035	0.620 **	0.002
PC 17:0/18:1	−0.263	0.131	0.398 *	0.041	0.120	0.307	0.670 **	0.001
PC 35:3	−0.414 *	0.035	0.277	0.118	0.428 *	0.030	0.636 **	0.001
PC 16:0/18:0	−0.272	0.123	0.571 **	0.004	0.292	0.106	0.689 **	0.000
PC 18:0/20:2	−0.245	0.149	0.343	0.069	0.462 *	0.020	0.651 **	0.001
LPC 26:0	−0.329	0.078	0.575 **	0.004	0.243	0.151	0.751 **	0.000
PC 14:0/18:3	−0.389 *	0.045	0.459 *	0.021	0.514 *	0.010	0.564 **	0.005
PC 18:0/18:1	−0.216	0.181	0.509 *	0.011	0.314	0.089	0.693 **	0.000
PC 19:0/18:2	−0.302	0.098	0.394 *	0.043	0.549 **	0.006	0.609 **	0.002
LPC 20:3	−0.216	0.180	0.287	0.110	0.290	0.107	0.577 **	0.004
LPC 15:0	−0.348	0.066	0.257	0.137	0.242	0.152	0.605 **	0.002
SUM LPC	−0.395 *	0.042	0.407 *	0.037	0.528 **	0.008	0.733 **	0.000

* for *p* values < 0.05 and ** for *p* values < 0.01.

**Table 4:** Association between adiposomal lysophosphatidylcholine and metabolic and inflammatory biomarkers.

	FPI		FPG		HOMA_IR		HDL-C		IL6			CRP
	r	*p*-Value	r	*p*-Value	r	*p*-Value	r	*p*-Value	r	*p*-Value	r	*p*-Value
LPC 17:0	−0.406 *	0.038	0.114	0.317	−0.252	0.142	0.270	0.125	−0.478 *	0.017	−0.520 **	0.009
LPC 18:0	−0.453 *	0.022	0.023	0.462	−0.310	0.092	0.216	0.181	−0.490 *	0.014	−0.518 **	0.010
LPC 18:0	−0.458 *	0.021	0.024	0.460	−0.313	0.090	0.220	0.175	−0.494 *	0.013	−0.523 **	0.009
LPC 24:0	−0.556 **	0.005	−0.144	0.272	−0.443 *	0.025	0.270	0.125	−561 **	0.005	−0.507 *	0.011
LPC 18:1	−0.398 *	0.041	0.082	0.366	−0.238	0.156	0.208	0.189	−0.473 *	0.017	−0.512 *	0.010
LPC 17:1	−0.396 *	0.042	0.098	0.341	−0.243	0.151	0.260	0.134	−0.494 *	0.013	−0.494 *	0.013
LPC 20:2	−0.455 *	0.022	0.015	0.474	−0.325	0.081	0.275	0.120	−0.522 **	0.009	−0.484 *	0.015
LPC 18:2	−0.476 *	0.017	−0.020	0.466	−0.351	0.065	0.383 *	0.048	−0.469 *	0.019	−0.558 **	0.005
PC 18:2/22:6	−0.525 **	0.009	0.058	0.404	−0.357	0.061	0.321	0.084	−0.379 *	0.050	−0.649 **	0.001
PC 18:2/20:4	−0.538 **	0.007	−0.064	0.395	−0.412 *	0.036	0.253	0.141	−0.523 **	0.009	−0.533 **	0.008
LPC 20:0	−0.501 *	0.012	−0.029	0.451	−0.362	0.058	0.188	0.213	−0.539 **	0.007	−0.568 **	0.004
LPC 19:0	−0.509 *	0.011	−0.011	0.482	−0.369	0.055	0.305	0.095	−0.604 **	0.002	−0.504 *	0.012
PC 36:3	−0.515 *	0.010	−0.060	0.401	−0.402 *	0.039	0.250	0.143	−0.538 **	0.007	−0.539 **	0.007
PC 18:1/18:3	−0.507 *	0.011	−0.093	0.348	−0.406 *	0.038	0.222	0.174	−0.565 **	0.005	−0.501 *	0.012
PC 18:2/18:2	−0.505 *	0.012	−0.105	0.329	−0.410 *	0.036	0.204	0.194	−0.572 **	0.004	−0.489 *	0.014
PC 16:1/18:2	−0.524 **	0.009	−0.074	0.379	−0.410 *	0.036	0.411 *	0.036	−0.562 **	0.005	−0.466 *	0.019
LPC 24:1	−0.411 *	0.036	0.016	0.473	−0.263	0.131	0.207	0.190	−0.635 **	0.001	−0.398 *	0.041
PC 18:1/18:2	−0.558 **	0.005	−0.191	0.210	−0.480 *	0.016	0.296	0.102	−0.625 **	0.002	−0.472 *	0.018
PC 18:1/22:5	−0.475 *	0.017	−0.041	0.433	−0.350	0.065	0.625 **	0.002	−0.540 **	0.007	−0.459 *	0.021
LPC 22:0	−0.205	0.193	0.276	0.120	−0.004	0.493	0.017	0.471	−0.669 **	0.001	−0.339	0.072
PC 17:0/18:2	−0.514 *	0.010	−0.161	0.249	−0.454 *	0.022	0.354	0.063	−0.513 *	0.010	−0.451 *	0.023
PC 17:0/18:1	−0.429 *	0.030	−0.017	0.471	−0.316	0.087	0.275	0.121	−0.384 *	0.047	−0.483 *	0.016
PC 35:3	−0.480 *	0.016	−0.108	0.325	−0.397 *	0.042	0.288	0.109	−0.553 **	0.006	−0.406 *	0.038
PC 16:0/18:0	−0.576 **	0.004	−0.122	0.304	−0.460 *	0.021	0.159	0.251	−0.376	0.051	−0.546 **	0.006
PC 18:0/20:2	−0.477 *	0.017	−0.118	0.310	−0.421 *	0.032	0.256	0.138	−0.468 *	0.019	−0.438 *	0.027
LPC 26:0	−0.497 *	0.013	−0.052	0.413	−0.339	0.072	0.517 **	0.010	−0.557 **	0.005	−0.616 **	0.002
PC 14:0/18:3	−0.465 *	0.019	−0.115	0.315	−0.367	0.056	0.230	0.165	−0.387 *	0.046	−0.468 *	0.019
PC 18:0/18:1	−0.501 *	0.012	−0.129	0.293	−0.418 *	0.033	0.231	0.163	−0.404 *	0.038	−0.556 **	0.005
PC 19:0/18:2	−0.566 **	0.005	−0.207	0.190	−0.510 *	0.011	0.187	0.215	−0.487 *	0.015	−0.457 *	0.021
LPC 20:3	−0.380 *	0.049	−0.007	0.489	−0.290	0.108	0.175	0.230	−0.396 *	0.042	−0.426 *	0.031
LPC 15:0	−0.429 *	0.029	0.041	0.431	−0.318	0.086	0.386 *	0.047	−0.365	0.057	−0.479 *	0.016
SUM LPC	−0.574 **	0.004	−0.130	0.292	−0.469 *	0.019	0.301	0.098	−0.609 **	0.002	−0.552 **	0.006

* for *p* values < 0.05 and ** for *p* values < 0.01.

**Table 5:** Association between adiposomal ceramides and anthropometric measurements.

	BMI		Fat %		Lean %	
	r	*p*-Value	r	*p*-Value	r	*p*-Value
Cer d37:0	0.410 *	0.036	0.445 *	0.025	−0.516 **	0.010
Cer d39:0	0.394 *	0.043	0.564 **	0.005	−0.639 **	0.001
Cer d41:0	0.421 *	0.032	0.563 **	0.005	−0.628 **	0.002
Cer d18:0/23:0	0.421 *	0.032	0.563 **	0.005	−0.628 **	0.002
Cer d40:0	0.429 *	0.030	0.613 **	0.002	−0.668 **	0.001
Cer d18:0/22:0	0.429 *	0.030	0.613 **	0.002	−0.668 **	0.001
Cer t42:0	0.428 *	0.030	0.606 **	0.002	−0.662 **	0.001
Cer d18:0/24:0	0.395 *	0.042	0.600 **	0.003	−0.652 **	0.001
Cer d42:0	0.363	0.058	0.608 **	0.002	−0.654 **	0.001
Cer d18:0/18:0	0.410 *	0.036	0.421 *	0.032	−0.497 *	0.013
Cer d38:0	0.385 *	0.047	0.542 **	0.007	−0.612 **	0.002
Cer t40:0	0.370	0.054	0.537 **	0.007	−0.612 **	0.002
Cer t43:0	0.394 *	0.043	0.540 **	0.007	−0.605 **	0.002
Cer d43:0	0.353	0.063	0.556 **	0.005	−0.590 **	0.003
Cer d18:1/25:0	0.498 *	0.013	0.527 **	0.009	−0.542 **	0.007
Cer d19:1/24:0	0.502 *	0.012	0.523 **	0.009	−0.537 **	0.007
Cer d18:2/24:0	0.475 *	0.017	0.441 *	0.026	−0.496 *	0.013
SUM Cer	0.273	0.123	0.427*	0.030	−0.470 *	0.018

* for *p* values < 0.05 and ** for *p* values < 0.01.

**Table 6:** Association between adiposomal ceramides and cardiovascular risk.

	SBP		FMD		FID		IL6	
	r	*p*-Value	r	*p*-Value	r	*p*-Value	r	*p*-Value
Cer d37:0	0.224	0.171	−0.328	0.079	−0.483 *	0.016	0.483 *	0.015
Cer d39:0	0.386 *	0.046	−0.383 *	0.048	−0.593 **	0.003	0.730 **	0.000
Cer d41:0	0.393 *	0.043	−0.334	0.075	−0.595 **	0.003	0.670 **	0.001
Cer d18:0/23:0	0.393 *	0.043	−0.334	0.075	−0.595 **	0.003	0.670 **	0.001
Cer d40:0	0.429 *	0.030	−0.362	0.059	−0.641 **	0.001	0.768 **	0.000
Cer d18:0/22:0	0.429 *	0.030	−0.362	0.059	−0.641 **	0.001	0.768 **	0.000
Cer t42:0	0.421 *	0.032	−0.365	0.057	−0.636 **	0.001	0.767 **	0.000
Cer d18:0/24:0	0.503 *	0.012	−0.348	0.066	−0.623 **	0.002	0.771 **	0.000
Cer d42:0	0.596 **	0.003	−0.339	0.072	−0.633 **	0.001	0.803 **	0.000
Cer d18:0/18:0	0.204	0.194	−0.320	0.084	−0.458 *	0.021	0.427 *	0.030
Cer d38:0	0.365	0.057	−0.394 *	0.043	−0.561 **	0.005	0.711 **	0.000
Cer t40:0	0.384 *	0.047	−0.404 *	0.039	−0.553 **	0.006	0.712 **	0.000
Cer t43:0	0.382 *	0.048	−0.314	0.089	−0.569 **	0.004	0.647 **	0.001
Cer d43:0	0.485 *	0.015	−0.290	0.107	−0.573 **	0.004	0.758 **	0.000
Cer d18:1/25:0	0.443 *	0.025	−0.514 *	0.010	−0.516 **	0.010	0.314	0.089
Cer d19:1/24:0	0.434 *	0.028	−0.507 *	0.011	−0.513 *	0.010	0.309	0.093
Cer d18:2/24:0	0.455 *	0.022	−0.560 **	0.005	−0.533 **	0.008	0.339	0.072
SUM Cer	0.453 *	0.022	−0.294	0.104	−0.470 *	0.018	0.704 **	0.000

* for *p* values < 0.05 and ** for *p* values < 0.01.

## Data Availability

The original contributions presented in the study are included in the article/supplementary material, further inquiries can be directed to the corresponding author/s.

## Supplementary Materials

The following supporting information can be downloaded at: https://www.mdpi.com/article/10.3390/metabo14080464/s1, Table S1: Adiposomal LPC/PC levels in obese and lean, healthy subjects and Table S2: Adiposomal levels of ceramides and other lipids in obese and lean, healthy subjects.
