# Supplementary material for: Comparison of Adiposomal Lipids between Obese and Non-Obese Individuals
Source: Metabolites. 2024 Aug 21;14(8):464. doi: 10.3390/metabo14080464 (PMC11356626; doi:10.3390/metabo14080464)
Supplement: Supplementary file 1 [file metabolites-14-00464-s001.zip › metabolites-3115356-supplementary.pdf]

Supplementary Table S1. Adiposomal LPC/PC levels in obese and lean, healthy subjects.

Compound	Q value	Log FC (Obese/Lean)	Regulation	Lipi d Class	Mass	Retention Time	Formula
PC 18:2/18:2	0.020	-3.27	down	PC	781.56	7.76	C44 H80 N O8 P
PC 18:1/18:3	0.020	-3.12	down	PC	781.56	7.76	C44 H80 N O8 P
PC 36:3	0.020	-3.08	down	PC	783.58	7.75	C44 H82 N O8 P
PC 14:0/18:3	0.039	-2.71	down	PC	727.51	6.47	C40 H74 N O8 P
PC 16:1/18:2	0.020	-2.45	down	PC	755.55	7.41	C42 H78 N O8 P
LPC 19:0	0.020	-2.23	down	LPC	537.38	4.28	C27 H56 N O7 P
LPC 20:0	0.020	-2.22	down	LPC	551.39	4.72	C28 H58 N O7 P
PC 18:2/20:4	0.020	-2.18	down	PC	805.56	7.6	C46 H80 N O8 P
PC 18:2/22:6	0.020	-2.09	down	PC	829.56	7.39	C48 H80 N O8 P
PC 35:3	0.037	-2.02	down	PC	769.56	8.1	C43 H80 N O8 P
LPC 18:2	0.020	-2.01	down	LPC	519.33	2.27	C26 H50 N O7 P
LPC 20:2	0.020	-1.95	down	LPC	547.36	3.25	C28 H54 N O7 P
LPC 20:3	0.042	-1.95	down	LPC	545.35	2.62	C28 H52 N O7 P
LPC 17:1	0.020	-1.92	down	LPC	507.33	2.43	C25 H50 N O7 P
LPC 18:1	0.020	-1.88	down	LPC	521.35	2.95	C26 H52 N O7 P
LPC 24:0	0.020	-1.78	down	LPC	607.46	6.53	C32 H66 N O7 P
LPC 18:0	0.020	-1.77	down	LPC	523.36	3.56	C26 H54 N O7 P
PC 18:1/22:5	0.023	-1.77	down	PC	833.59	9.11	C48 H84 N O8 P
PC 18:0/20:2	0.037	-1.76	down	PC	813.63	12.27	C46 H88 N O8 P
PC 18:1/18:2	0.020	-1.75	down	PC	783.58	8.92	C44 H82 N O8 P
LPC 22:0	0.023	-1.71	down	LPC	579.43	5.53	C30 H62 N O7 P
LPC 17:0	0.020	-1.62	down	LPC	509.35	3.28	C25 H52 N O7 P
PC 17:0/18:1	0.037	-1.61	down	PC	773.59	11.39	C43 H84 N O8 P
LPC 15:0	0.043	-1.61	down	LPC	481.31	2.22	C23 H48 N O7 P
LPC 24:1	0.020	-1.54	down	LPC	605.44	5.57	C32 H64 N O7 P
PC 17:0/18:2	0.037	-1.53	down	PC	771.58	9.65	C43 H82 N O8 P
PC 16:0/18:0	0.037	-1.48	down	PC	761.59	11.99	C42 H84 N O8 P
PC 18:0/18:1	0.039	-1.42	down	PC	787.61	12.11	C44 H86 N O8 P
PC 19:0/18:2	0.041	-1.4	down	PC	799.61	11.92	C45 H86 N O8 P
LPC 26:0	0.038	-1.25	down	LPC	635.49	7.83	C34 H70 N O7 P

LPC, Lysophosphatidylcholine; PC, phosphatidyl choline, PE, phosphatidyl ethanolamine

Supplementary Table S2. Adiposomal levels of ceramides and other lipids in obese and lean, healthy subjects.

Compound	Q value	Log FC (Obese/Lean)	Regulation	Lipid Class	Mass	Retention Time	Formula
TG 16:0/16:0/16:0	0.0002	4.64	up	TG	806.74	15.78	C51 H98 O6
Cer d37:0	0.020	3.76	up	Cer	581.57	12.33	C37 H75 N O3
Cer d18:0/18:0	0.020	2.9	up	Cer	567.56	12.31	C36 H73 N O3
SM d36:0	0.023	2.76	up	SM	732.61	10.59	C41 H85 N2 O6 P
Cer d39:0	0.020	2.05	up	Cer	609.6	12.79	C39 H79 N O3
SM d40:0	0.031	2.01	up	SM	788.68	12.78	C45 H93 N2 O6 P
Cer d38:0	0.023	1.93	up	Cer	595.59	12.79	C38 H77 N O3
Cer d41:0	0.020	1.92	up	Cer	637.64	13.34	C41 H83 N O3
Cer d18:0/23:0	0.020	1.92	up	Cer	637.64	13.34	C41 H83 N O3
Cer d40:0	0.020	1.86	up	Cer	623.62	13.16	C40 H81 N O3
Cer d18:0/22:0	0.020	1.86	up	Cer	623.62	13.16	C40 H81 N O3
Cer t40:0	0.023	1.86	up	Cer	655.61	12.79	C40 H81 N O5
Cer d18:0/24:0	0.020	1.84	up	Cer	651.65	13.53	C42 H85 N O3
Cer t42:0	0.020	1.77	up	Cer	683.64	13.16	C42 H85 N O5
Cer d42:0	0.020	1.77	up	Cer	651.65	13.53	C42 H85 N O3
Cer t43:0	0.024	1.65	up	Cer	697.66	13.34	C43 H87 N O5
Cer d43:0	0.029	1.65	up	Cer	665.67	13.53	C43 H87 N O3
Cer d18:1/25:0	0.042	1.62	up	Cer	663.65	13.54	C43 H85 N O3
Cer d19:1/24:0	0.043	1.60	up	Cer	663.65	13.54	C43 H85 N O3
Cer d18:2/24:0	0.043	1.59	up	Cer	647.62	13.06	C42 H81 N O3
CE 18:1 (d7)	0.028	1.28	up	CE	657.64	16.65	C45 H71 D7 O2
Cer d26:0	0.048	-0.32	down	Cer	523.36	3.8	C26 H54 N O7 P
Cer d18:1/24:1	0.049	-0.42	down	Cer	647.62	13.06	C42 H81 N O3
FA 21:0	0.020	-1.34	down	FA	326.32	5.85	C21 H42 O2
FA 22:0	0.020	-1.37	down	FA	340.33	6.43	C22 H44 O2
PE 38:5	0.020	-1.72	down	PE	765.54	8.92	C43 H76 N O8 P

Cer, ceramide; FA, fatty acid; TG, triglyceride; SM, sphingomyelin
